# A Case Report of a Metastatic Gastrointestinal Stromal Tumor Occurring in Femur

**DOI:** 10.1155/2011/926179

**Published:** 2011-09-22

**Authors:** Chang-kun Zheng, Wu-Sheng Kan, Peng Li

**Affiliations:** Department of Orthopaedics, Pu Ai Hospital of Tongji Medical College of Huazhong University of Science and Technology, Hanzheng Street 473, Hubei Province Wuhan 430033, China

## Abstract

Gastrointestinal stromal tumors (GISTs) are mesenchymal neoplasms that most commonly affect the stomach or small intestine, but can occur anywhere throughout the gastrointestinal tract. To the best of our knowledge, few cases have been reported in the literature about the femur metastasis of GIST. This paper describes a metastasis of a gastrointestinal stromal tumour (GIST) to the femur in a 62-year-old male, 2 years after treatment for a gastric primary. There were no signs of tumor recurrence at followup after 12 mo. This case suggests that the femur can be a potential metastatic site of GIST.

## 1. Introduction

Gastrointestinal stromal tumour (GIST) is a rare disease. The crude incidence rates of this soft tissue sarcoma are 1.5 individuals per 100,000 persons and year [1]. GIST most commonly arises from the stomach and accounts for about 1% of gastric malignancies [2]. Liver and peritoneal metastatic lesions are often observed, but femur metastasis is rarely found in GIST. Metastases are located intra-abdominally in more than 90% [3]. There are few reports about the femur metastasis of GIST in the literature. Here we report one interesting GIST case with femur metastases 2 years after distal gastrectomy due to gastric GIST.

## 2. Report

**Figure 1:**
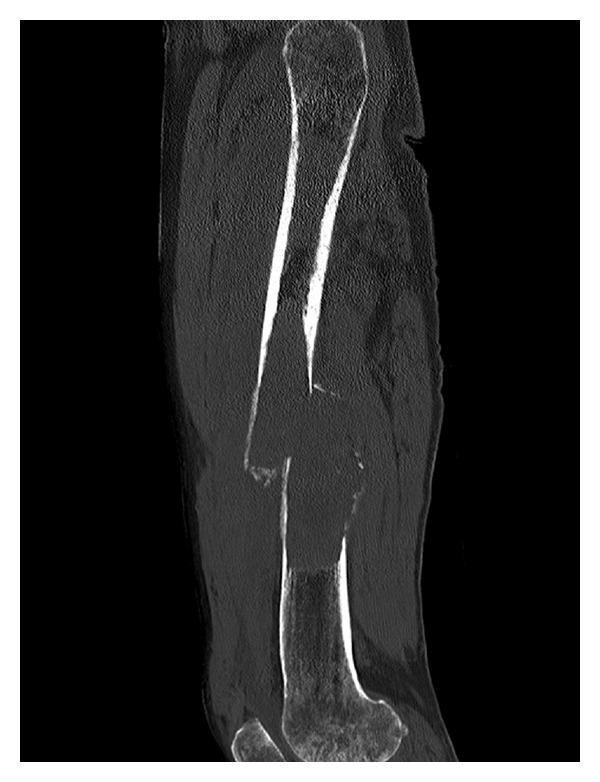


**Figure 2:**
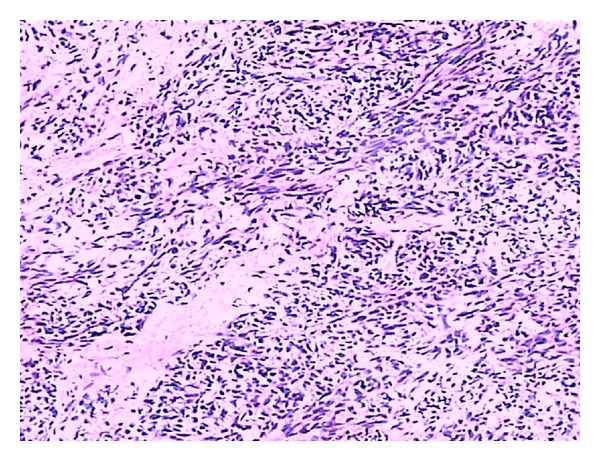


**Figure 3:**
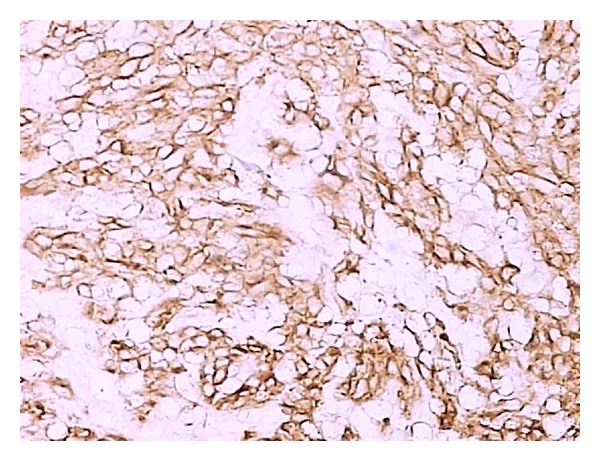


**Figure 4:**
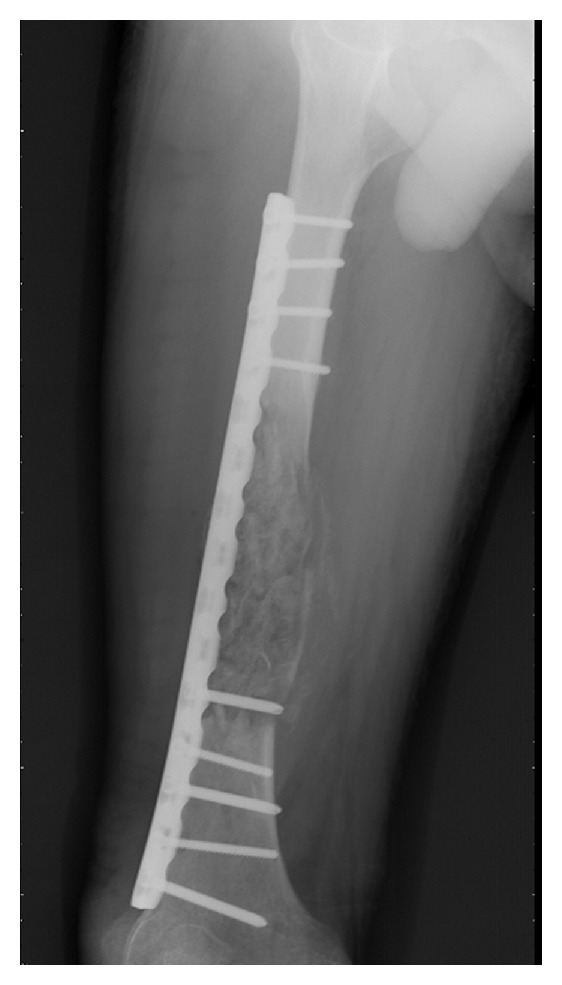


Gastrointestinal stromal tumor (GIST) is the most common mesenchymal tumor in the alimentary tract. Here we report an unusual case of gastric GIST with femur metastasis. A 62-year-old man came to the clinic on January 16th, 2010, complaining of his feeling of left thigh pain. X-ray demonstrated the left femur pathologic fracture. He received distal gastrectomy, perigastric lymphadenectomy in February 2008 for gastric GIST. Physical examination revealed a 4 cm thickening which was located in the left femur. Computed tomography (CT) revealed an 8.5 cm × 4 cm × 3.5 cm low density mass (CT value = 69.7 HU) (Figure 1). A complete focus of tumor excision of the mass, bone graft, internal fixation was carried out for this patient on January 20th, 2010. Grossly, the surgical specimen consisted of a 9 cm × 4 cm × 3.5 cm large, jelly, whitish-gray. Microscopically, it was confluent and was infiltrated with spindle cells. These spindle cells were predominantly arranged in interweaving fascicles (Figure 2). Immunohistochemical stains demonstrated a strong positivity for all c-kit (CD117), CD34, Vimentin, and S-100 (Figure 3), which was consistent with the primary tumor. Gene sequence analysis showed that both the primary tumors and the resected tumor harbored deletion mutations affecting codons 557/558 of exon 11 in the KIT. After the operation, the patient took imatinib 400 mg/d for 1 year. And there was no sign of tumor recurrence during 12 mo of followup (Figure 4).

## 3. Discussion

GISTs are the most common mesenchymal tumours of the gastrointestinal tract defined by expression of c-KIT (CD 117). The paper describes the diagnosis and therapy of a GIST metastasis to the femur. The case we reported herein is a high-grade GIST according to the Bucher grading system [3]. Unlike gastric adenocarcinoma, femur metastasis of GIST is unusual. In this case, perigastric lymphadenectomy was performed and no metastasis was observed in all of 19 lymph nodes at the first operation, no liver metastasis was detected. With regards to a proposal on a recent histopathological and prognostic tumour classification of GIST, patients with distant metastasis constitute a separate risk group. In these cases, the size of the tumour and the mitotic index are irrelevant factors in estimating crude survival [4]. It is likely that some patients benefit from resecting recurrent tumour in cases with isolated local or metastatic disease, given the chance to resect this tumour completely. The high rate of local and distant tumour recurrence underlines the necessity for additional therapies.

At present, surgery remains the mainstay of treatment in GIST patients with isolated resectable GIST. Treatment with imatinib was introduced for patients with metastatic disease as an adjunct to surgery or in cases with nonresectable tumour. The persistent use of imatinib was necessary for this patient after complete resection of primary gastric and metastatic GISTs. After his second operation, the patient started imatinib treatment and survived without recurrence till now. Imatinib proved to be effective in terms of overall survival and arrest of tumour progression [5, 6].

In conclusion, GIST can involve the femur as a metastatic site and further studies are necessary to clarify the mechanism of this metastasis.

## References

[1] Mucciarini C, Rossi G, Bertolini F (2007). Incidence and clinicopathologic features of gastrointestinal stromal tumors. A population-based study. *BMC Cancer*.

[2] Miettinen M, Furlong M, Sarlomo-Rikala M, Burke A, Sobin LH, Lasota J (2001). Gastrointestinal stromal tumors, intramural leiomyomas, and leiomyosarcomas in the rectum and anus: a clinicopathologic, immunohistochemical, and molecular genetic study of 144 cases. *American Journal of Surgical Pathology*.

[3] Bucher P, Egger JF, Gervaz P (2006). An audit of surgical management of gastrointestinal stromal tumours (GIST). *European Journal of Surgical Oncology*.

[4] Demetri GD, Von Mehren M, Blanke CD (2002). Efficacy and safety of imatinib mesylate in advanced gastrointestinal stromal tumors. *The New England Journal of Medicine*.

[5] Fletcher CDM, Berman JJ, Corless C (2002). Diagnosis of gastrointestinal stromal tumors: a consensus approach. *Human Pathology*.

[6] Verweij J, Casali PG, Zalcberg J (2004). Progression-free survival in gastrointestinal stromal tumours with high-dose imatinib: randomised trial. *The Lancet*.

